# Fovea-Periphery Axis Symmetry of Surround Modulation in the Human Visual System

**DOI:** 10.1371/journal.pone.0057906

**Published:** 2013-02-28

**Authors:** Lauri Nurminen, Markku Kilpeläinen, Simo Vanni

**Affiliations:** 1 Brain Research Unit, O.V. Lounasmaa Laboratory, Aalto University, Espoo, Finland; 2 Institute of Behavioural Sciences, University of Helsinki, Helsinki, Finland; 3 Advanced Magnetic Imaging Centre, AALTO NEUROIMAGING, Aalto University, Espoo, Finland; University of Minnesota, United States of America

## Abstract

A visual stimulus activates different sized cortical area depending on eccentricity of the stimulus. Here, our aim is to understand whether the visual field size of a stimulus or cortical size of the corresponding representation determines how strongly it interacts with other stimuli. We measured surround modulation of blood-oxygenation-level-dependent signal and perceived contrast with surrounds that extended either towards the periphery or the fovea from a center stimulus, centered at 6° eccentricity. This design compares the effects of two surrounds which are identical in visual field size, but differ in the sizes of their cortical representations. The surrounds produced equally strong suppression, which suggests that visual field size of the surround determines suppression strength. A modeled population of neuronal responses, in which all the parameters were experimentally fixed, captured the pattern of results both in psychophysics and functional magnetic resonance imaging. Although the fovea-periphery anisotropy affects nearly all aspects of spatial vision, our results suggest that in surround modulation the visual system compensates for it.

## Introduction

Perception of a visual target relies strongly on the spatiotemporal context in which the target appears. For example, surrounding texture reduces perceived contrast [1], [2], [3], [4], [5] and raises contrast detection thresholds [3], [6] of embedded targets. Similar phenomenon occurs in single cells of the primary visual cortex, where an appropriate stimulus outside the classical receptive field reduces firing rate elicited by a stimulus within the classical receptive field [7], [8], [9], [10], [11], [12], [13], [14], [15]. In addition to suppression, spatial context may also increase firing rate of single cells [16], [17], [18], perceived contrast [1], [5], [19], [20] and contrast sensitivity [21], [22], [23]. These context mediated effects are commonly termed surround modulation.

Functional magnetic resonance imaging (fMRI) has shown that surround modulation emerges also at the level of large neuronal populations [24], [25], [26], [27], [28], [29]. In fact, our recent study showed that after the retinotopic coverage of a voxel is accounted for, surround modulation of V1 blood oxygenation-level dependent (BOLD) responses agrees fairly well with psychophysics [24].

In the primary visual cortex, surround modulation results most likely from the interplay between thalamic inputs, intra-areal horizontal connections and feedback from the extra-striate cortices [7], [30], [31], [32], [33]. The horizontal and feedback connections mediate short-range effects whereas feedback connections mediate long-range suppression and facilitation [17], [33]. Horizontal connections extend symmetrically the same distance towards the foveal and peripheral visual field representations, whereas the feedback projection is asymmetric in visual field coordinates [7]. Although the evidence concerning the visual field symmetry of the connections underlying surround modulation in V1 neurons is inconclusive, current models posit that the modulatory region is symmetric in the visual field [9], [34]. For example, for a receptive field in the peripheral visual field, the modulatory surround region would symmetrically extend the same distance towards the fovea and periphery. In addition to the neuronal receptive field models, the visual field symmetry assumption is also implicit in some of the retinotopic mapping techniques [35], [36]. However, the modulatory surround region could, in principle, be symmetric in the cortex and thus asymmetric in the visual field, but previous studies have not carefully investigated such possibility. Dissecting these alternatives is possible with an experiment in which the visual field sizes of two surrounds are identical, but cortical magnification renders their cortical representations different.

Petrov, Popple, & McKee [37] studied surround modulation of contrast detection with stimuli that were appropriate for revealing whether surround’s cortical or visual field size determines strength of surround suppression. They used hemi-annular surrounds that extended either towards periphery or the fovea from the peripheral target. Although the surrounds in the study of Petrov et al. [37] must have had entirely different sized cortical representations, they produced essentially equal suppression strengths. Unfortunately, the possibility of ceiling effect cannot be ruled out in the study because their surround width was 6 times the target wavelength and Cannon and Fullenkamp [38] have specifically shown that suppression strength saturates at this surround width. Increasing the surround width beyond 6 cycles does not increase suppression strength when the surround is near the center [38]. Therefore, it is possible that Petrov et al. [37] have missed possible differences between foveal and peripheral surrounds.

Here, we measured surround suppression of perceived contrast and V1 BOLD signals in the presence of two types of surrounds, one extending towards the fovea and one towards the periphery from the center. The rationale of this design is that although the two surrounds are identical in visual field their cortical representations markedly differ. To avoid ceiling effects which could mask subtle differences between the two surround types, we varied the width of a gap separating the center and the surround. Suppression strengths were equal between the surround types both in psychophysics and fMRI, which suggests that the visual field size of the surround determines suppression strength. A modeled population of single neuron responses, with fixed parameters, captured the overall pattern of the psychophysical and the fMRI results. The results of our second fMRI experiment suggested that suppression strengths were equal because the surrounds produced equally strong signals in the retinotopic representation of the center, and because the remaining differences were attenuated when the surrounds were combined with the center. This study suggests that the human visual system compensates for the fovea-periphery anisotropy in surround modulation.

## Methods

### Ethics Statement

The subjects gave their written informed consent and the ethical committee of the Hospital District of Helsinki and Uusimaa approved the study.

### Psychophysics

#### Subjects

Five subjects participated in the psychophysical experiments. Subjects S1 and S3 were authors of this study, subjects S2 and S4 were naïve and inexperienced. The observers had normal or corrected-to-normal visual acuity.

#### Stimuli

In all experiments, Michelson contrast, spatial and temporal frequency of the sinusoidal gratings was 30%, 1cpd and 5 Hz, respectively, and grating orientation was vertical. The eccentricity was always 6° and the stimuli were displayed on the horizontal meridians. The mean luminance of the stimulus and the unmodulated background was 40 cd/m^2^.

In the *area summation* experiment, the pedestal stimulus was a circular grating patch. Nine pedestal diameters were used (1, 1.25, 1.5, 1.75, 2, 2.5, 3.5, 4.5 and 7.5°). Pedestal diameter 1.25° was not used in subject S2. The diameters refer to the plateau diameters of the 10^th^ order butterworth window. The superimposed target was otherwise similar than the pedestal gratings, but within a 36^th^ order butterworth window with a 1° plateau diameter.

**Figure 1:**
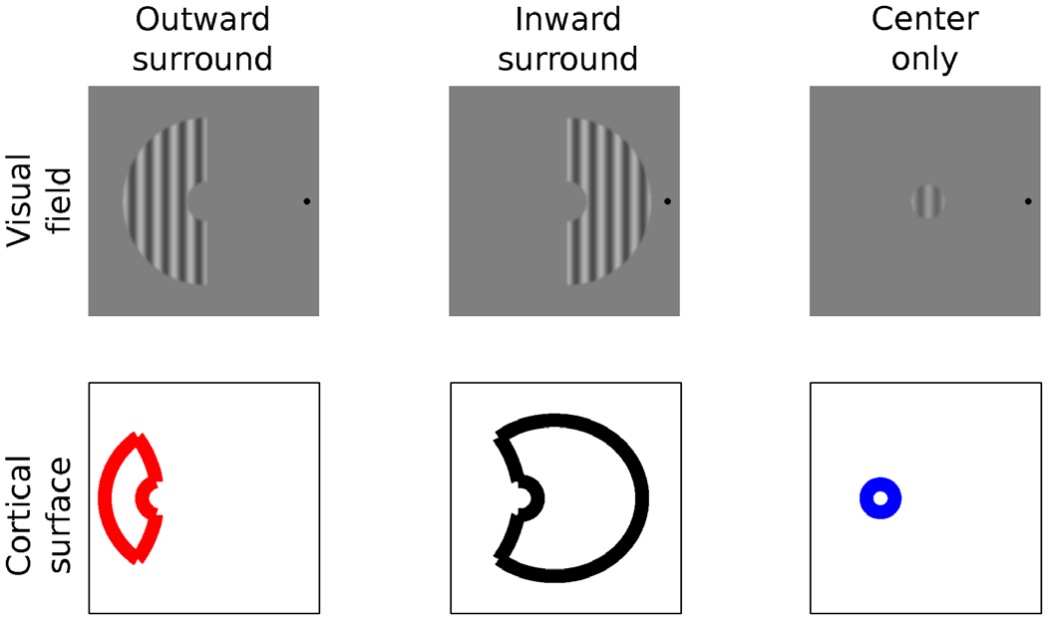
The stimuli and their corresponding cortical representations. Upper row illustrates the two surrounds types and the center used in this study. The lower row depicts canonical cortical surface representations of the same stimuli computed using the Schwarz [47] formula. The red color indicates here and below the *outward* surround and black the *inward* surround.

In the *surround modulation* experiment, the test stimuli consisted of gratings in center-surround configuration (Figure 1). Diameter of the center was 1.8°. The outer radius of the surround hemi-annulus was always 5 degrees. Five gap sizes between the center and surround were used (0.1, 0.35, 0.6, 1.1 and 2.1°). Sharp edges of the stimuli were smoothed with a Gaussian filter (SD 6 pixels). We adopt terminology from Petrov et al. [37] and term the surround extending towards the fovea *inward* surround and the one extending towards the periphery the *outward* surround. The comparison stimulus was identical with the test center, except for the contrast which was varied.

#### Apparatus

The stimuli were created with Matlab™ (Natick, MA, USA) and displayed with Cambridge Research System’s (Kent, UK) VisaGe graphics card providing 14-bits gray-scale resolution. The monitor was a 22 inches Mitsubishi Diamond Pro 2070 CRT with 800×600 pixels (39.0×29.2 cm) resolution. The luminance output of the CRT was linearized. The viewing distance was fixed to 90 cm with a chin rest.

#### Procedure

In the *area summation* experiment, the pedestals were simultaneously displayed on the right and left sides of the fixation. One of the pedestal gratings also contained the superimposed target. The side of the target was randomized for each trial. Subjects indicated with a button press on which side the target was. Auditory feedback was given upon incorrect responses. Temporal design of the experiment was as follows; fixation cross first appeared on the screen for 300 ms and then blank screen was shown for 300 ms. Next the pedestals with and without the target were simultaneously displayed for 300 ms. After the stimulus presentation, subjects indicated with a button press the side on which the target appeared to be on. The button press initiated a new trial. Target contrast was lowered after the subject correctly identified the target location in three successive trials and increased after each error. This standard 3-1 staircase procedure yields the 75% correct threshold. The step contrast was 7% for the initial four reversals and 1.6% for the last six reversals. Thresholds were calculated from the final six reversals. In the RESULTS the mean ± s.e.m. of the final staircase reversals is reported.

In the *surround modulation* experiment, test and comparison stimuli were simultaneously displayed on the right and left sides of the fixation. The positions were randomized in each trial. Subjects were instructed to compare contrasts of the test center and the comparison stimulus and to ignore the surround. Temporal sequence of a trial was as follows. First, the fixation cross appeared on the screen for 300 ms and then blank screen was shown for 300 ms. Then, the fixation cross, the test and the comparison stimulus were simultaneously displayed for 500 ms. After the stimulus presentation, subjects indicated with a button press the side in which contrast of the center appeared higher. The button press initiated the next trial.

Contrast of the comparison stimulus was controlled by a randomly interleaved 1-1 double-staircase protocol. Initially, the contrasts of the two staircases were set clearly above and below the perceived contrast of the test center. Contrast of the comparison stimulus was lowered if it appeared higher than the contrast of the test center. Otherwise the contrast was increased. The reversal contrast was recorded and the procedure continued until there were 6 reversals in both of the staircases. The first 2 reversals of each staircase were considered as practice and omitted from the calculation of the perceived contrast. The reported values are the means of the remaining staircase reversals. Measurements were repeated on two different days. In the Results, we report the mean ± s.e.m, in which the s.e.m is calculated from the four independent staircase estimates of the perceived contrast.

### fMRI

#### Subjects

Eight subjects (two females) participated in the first fMRI experiment and ten (one female) in the second. Seven of the subjects participated in both fMRI experiments. All subjects had normal or corrected-to-normal visual acuity. Data of one of the subjects in the first fMRI experiment was rejected because in this subject the suppression strengths deviated from the rest of the subject population by more than three standard deviations.

### Stimuli

#### fMRI experiment 1

The stimuli were identical to those used in the psychophysical surround modulation experiment except for the 4.44 Hz drift rate and spatial dimensions. Our previous study [24] showed that the summation area is larger in fMRI than in psychophysics. Thus the diameter of the center was increased to 3°. Three gap sizes were used (0.1, 0.6 and 1.8°).

#### fMRI experiment 2

In this experiment, only a 0.2° gap size was used. Center diameter was 2°. Contrast of the center was 20% and contrast of the surround was 40%. The second fMRI experiment included also conditions in which the surround stimuli were displayed alone. Drift direction and the orientation of all the stimulus parts changed after each drift-cycle. Orientations 0, 45, 90 and 135 degrees appeared in random order, but the same orientation was never displayed successively. Center and surround always had the same orientation and drifted in-phase to the same direction.

In the functional localizer runs the stimulus was spatially identical with the corresponding center stimulus and its contrast was 100%.

### Timing

#### fMRI experiment 1

A measurement session consisted of nine experimental runs, two localizer runs and one run for obtaining a T1-weighted structural image. In the experimental runs the block length was 10.8 sec. The gap size and the side of the surround varied between the blocks. The same stimulus (e.g. center only) was presented throughout one block and different stimuli were presented in different blocks. The grating stimulus was drifting for the entire duration of the block and the drift direction reversed after every four cycles of drift. The block order remained the same within one run, but for each run the order was separately counterbalanced. The transitional effects between blocks were reduced by inserting one 1.8 sec of blank screen between the blocks. To reach stable magnetization there were no stimuli during the first 54 sec of each run. The blocks were repeated twice per run and 18 times in total. Duration of each run was 3 min 43 sec.

In the localizer runs the stimulus blocks and blank screen alternated with 50% duty cycle. Duration of the run was 3 min 36 sec.

#### fMRI experiment 2

A measurement session consisted of five experimental runs, two localizer runs and one run for structural image. Block length was 14.4 sec. The block order remained the same within one run, but for each run the order was separately counterbalanced. The transitional effects between blocks were reduced by inserting three time-points of blank screen between the blocks. In order to reach stable magnetization there were no stimuli during the first 54 sec of each run. The blocks were repeated three times per run and 15 times in total. Duration of each run was 6 min 22 sec.

In the localizer runs the stimulus blocks and blank screen alternated with 50% duty cycle. Duration of the run was 4 min 23 sec.

### Fixation Task

In both fMRI experiments, we used a fixation task that was adapted from Larsson, Landy and Heeger [39]. Subjects were instructed to pay attention to the stream of Z, N, L and T letters, which appeared on the center of the display. The letters appeared one at a time and always in the order specified above. The letters were updated every 225 ms. The letter sequence was looped throughout the presentation of a stimulus block. For each looped sequence 1-4 of the letters were randomly replaced by the letter X. The X letters were always separated by at least one of the other letters. At the end of the letter sequence subjects indicated the number of X letters they saw in the sequence, by pressing one of the four buttons in the response pad in their right hand. In addition to attention, this task ensures that subjects fixate, or they are not able to perform the demanding task.

### Display System

The stimuli were created using Matlab™ and their presentation was controlled with the Presentation™ (Neurobehavioral Systems Inc., Albany, CA, USA) software. The display was updated at 60 Hz. We used a 3-micromirror projector system X3™ (Christie Digital Systems Ltd., Cypress, CA, USA) to project the stimuli to the magnet room and into the bore. Gamma correction was used. The image was formed on a semitransparent plastic screen. In the first experiment the viewing distance was 43 cm and 34 cm in the second experiment. The resolution of the display was 1024×768 pixels (27.4 cm×20.5 cm in the first, and 28 cm×21 cm in the second experiment).

### Acquisition and Preprocessing

The fMRI data for the first experiment was acquired with a Siemens MAGNETOM Skyra 3T MRI (Siemens AB, Erlangen, Germany) scanner, equipped with 32-channel coil. The data for the second experiment was acquired with a General Electric Signa Hdxt 3.0T MRI (General Electric Medical Systems, Milwaukee, WI, USA) scanner, equipped with a 16-channel phased array coil. In both experiments we used spin-echo EPI because it provides superior spatial specificity compared to the conventional gradient-echo EPI [40], [41]. The 64×64 oblique acquisition matrix had 160 mm field-of-view and 2.5 mm slice thickness thus producing 2.5^3^ mm^3^ volume for a voxel. The repetition time was 1800 ms and echo time 70 ms. The slices were carefully positioned to cover the calcarine sulcus. There were 15 slices in the first experiment and 16 in the second.

Before the actual analyses, the DICOM-images were converted to NIFTI format, slice acquisition times were corrected and all volumes were realigned and resliced to the volume that was measured just before the structural volume [42]. The preprocessing steps were implemented with SPM8 software package (Wellcome trust center for neuroimaging, London, UK).

### Region of Interest Analysis

#### fMRI experiment 1

Two different ways for selecting the regions of interests were used. In the first, all the voxels in which the localizer produced statistically significant responses (t-test, family-wise error correction, p<0.05) and which were confined to the calcarine sulcus were analyzed. The cluster selected in this way was further projected to the unfolded and reconstructed cortical surface for confirmation of its location on the primary visual cortex. In the second, what we call voxel-of-interest analysis, the above threshold voxels were first projected to the reconstructed and unfolded 2D cortical surface and the analyses were computed on the one voxel, which was nearest to the geometrical center of the cluster.

#### fMRI experiment 2

The voxels in which the localizer produced statistically significant responses (t-test, family-wise error correction, p<0.05) and which were confined to the calcarine sulcus were analyzed. To confirm that the cluster was located on the primary visual cortex it was projected to unfolded cortical surface (see below). The analyses were further restricted to those voxels, in which the BOLD response to the simultaneous presentation of the center and the surround was less than to the center alone. This ensured that 1) our analysis concerns suppression instead of nonlinear summation 2) that saturation does not account for the suppression.

### Computing the Signal Changes

The BOLD percent signal-changes (% sc) were estimated with general linear model, implemented in SPM8. First, the time course of each stimulus condition was convolved with a canonical hemodynamic response function. Second, the coefficients associated with each condition were estimated. High-pass filter (128 sec cutoff) and noise autocorrelation estimates were included in the model. In addition to the stimulus conditions, each run included one coefficient for the mean signal. Third, the signal changes were obtained by dividing the stimulus related coefficients by the mean coefficient in that run, and multiplying the result by 100.

### Suppression Strength

Strength of psychophysical surround suppression and BOLD signal reduction was quantified as percentage reduction either in center’s perceived contrast or the BOLD response. In mathematical terms this is expressed as, suppression strength = 100* (C – CS)/C, in which C denotes perceived contrast or BOLD response to the center alone, and CS with the surround.

### Retinotopic Mapping and Surface Analysis

We used the multifocal technique [43] to map the borders of the early visual cortical areas. The original design was adapted to contain 24 stimulus regions in a temporal design that assured that neighboring regions were never simultaneously stimulated [44].

A structural volume with 1 mm×1 mm×1 mm resolution was obtained from each subject. Using this volume the border of white and gray matter was segmented with Freesurfer 5.0 software package [45], [46]. The structural volumes and the data for the retinotopic mappings were obtained in sessions that were separate from the main experiment.

### Model

Our previous model [24] was used for bridging the results from psychophysics and fMRI. Parameters of the model were strictly constrained by the preliminary area summation experiment. Source code of the model can be found on our web-page (http://ltl.tkk.fi/wiki/BRU/Vision_Systems_Neuroscience#Code).

**Figure 2:**
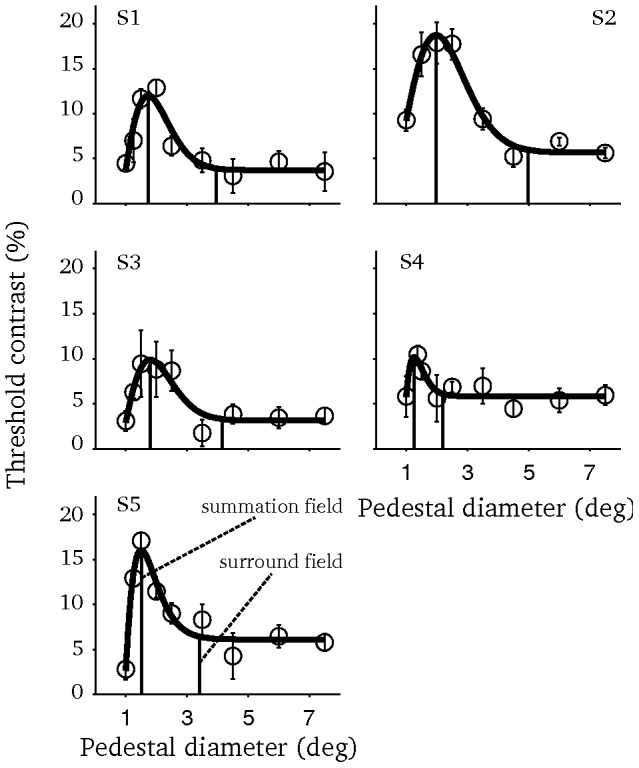
Area summation functions. Each panel depicts the data of one subject. Smooth lines represent the fitted difference-of-integrals of Gaussians function. Vertical dashed lines mark the summation and surround field sizes. Errorbars depict the s.e.m.

For each subject, threshold versus pedestal diameter functions were measured and fitted with difference-of-integrals of Gaussians functions (Figure 2). Three quantities were extracted from the fitted functions; summation field size is the pedestal diameter in which the function peaks, surround field size is the smallest pedestal diameter with which the threshold is within 5% of the threshold at the largest pedestal diameter, and suppression strength is the percentage reduction in threshold from the peak to the value at the largest pedestal diameter. Averaged over the subjects, summation field size was 1.71±0.12° (mean ± s.e.m), surround field size was 3.87±0.47° and the suppression strength was 64±5%.

Each stereotypical model neuron (N = 441) was described with a 2-dimensional variant of the difference-of-integrals of Gaussians receptive field model (Equation 1) [34].
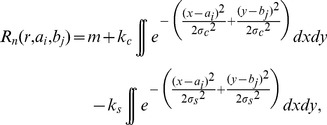
(1)where *σ_c_* = center spread, *k_c_* = center gain, *σ_s_* = surround spread, *k_s_* = surround gain, and *m* is a constant. The integrals in equation 1 were computed over the stimulus area. For the fMRI experiments, the model parameters were fixed to produce the mean summation and surround field sizes along with the suppression strength in the area summation experiment. The used parameter values were *σ_c_* = 0.48, *k_c = _*1.38, *σ_s = _*0.52, *k_s = _*1.10 and m = −0.12.

The receptive field center locations were calculated as follows: first the location of the stimulus center-point was projected to the cortical surface using Schwarz [47] formula, w = k*log(z+a), in which *w* is a complex number representing a point on unfolded cortical surface, *z* is the corresponding visual field location, *a* controls for the size of foveal representation and *k* is a scaling parameter. We used *a = *1 and *k* = 17, which produce the average magnification in human primary visual cortex. Next, the model neurons were evenly distributed on a 7.5 mm×7.5 mm lattice centered on the cortical representation of the stimulus center-point. The central 2.5 mm×2.5 mm corresponds to the modeled voxel and rest of the lattice is used for modeling the effect of point-spread. Finally, the lattice was projected back to the visual field using the inverse of the Schwarz [47] formula.

To model the technical point-spread of spin-echo EPI the central 2.5 mm×2.5 mm of the modeled cortex was set to one and the rest to zero. The lattice was then filtered in both dimensions with Cauchy-Lorentz function with half-width at half-maximum of 0.21 mm [48]. It is important here to make the distinction between the technical point-spread and the point-spread of the BOLD signal. After filtering, the modeled cortical patch was normalized to one and the response of each model neuron was weighted with the value in the corresponding location.

Finally, responses of the model neurons were summed for achieving the modeled voxel response. Because our previous study [24] showed that in V1 the suppression of BOLD signal is 2.7 times stronger than suppression of neuronal spike responses, the modeled BOLD signal reductions were multiplied by this factor.

To model surround suppression of perceived contrast, only one model neuron with receptive field centered on the stimulus was used. For each subject, the model parameters were individually fixed with the area summation experiment. Suppression strengths were not scaled.

## Results

### Surround Suppression of Perceived Contrast

The aim of this experiment was to find out whether cortical size or visual field size of the surround determines suppression strength. Two surrounds were used: the *inward* surround extended towards the fovea and the *outward* surround extended towards the periphery from the center, which was located at 6° eccentricity. Although the visual field representations of the surrounds were identical, their cortical representations were markedly different due to the non-linear cortical magnification factor.

**Figure 3:**
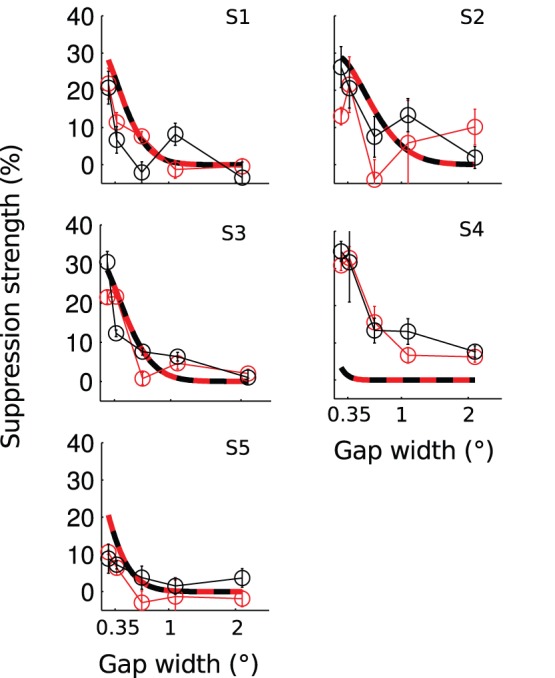
Surround suppression of perceived contrast as a function of the gap size. The different panels present data of different subjects. Connected data points mark the measured mean ± s.e.m. Smooth curves present the modeled suppression. Model parameters were fixed based on the area summation experiment for each subject separately. Red marks the *inward* surround condition and black the *outward*.

The main finding of this experiment was that the *inward* and the *outward* surrounds produced highly similar effects on perceived contrast of the center (Figure 3). Suppression was strong with small gap sizes and increasing the gap size decreased suppression strength. As the gap size was increased from 0.1 to 2.1°, the mean suppression strength decreased from 24.5±4.2% to 4.7±1.5% (paired two-tailed t-test, t(4) = 3.74, p<0.05) in the *inward* surround condition. For the *outward* surround, the corresponding decrease was from 18.7±4.2% to 3.5±2.1% (t(4) = 3.62, p<0.05). In all of the subjects and gap sizes, difference between the inward and the outward surrounds was statistically insignificant (paired two-tailed t-tests, family-wise error corrected, p>0.05).

With fixed set of parameters, our model captured the overall pattern of the data with reasonable accuracy (dashed lines in Figure 3). As the gap size was increased suppression strength decreased with a comparable slope in the model and the data. At the smallest gap size, the modeled suppression was somewhat stronger than the measured suppression. In subject S5 the model predictions did not match the data; in this subject summation field size in the preliminary area summation experiment was unexpectedly small and this difference prevailed also in a repeated area summation measurement.

### fMRI Experiment 1: Surround Induced BOLD Signal Reduction

**Figure 4:**
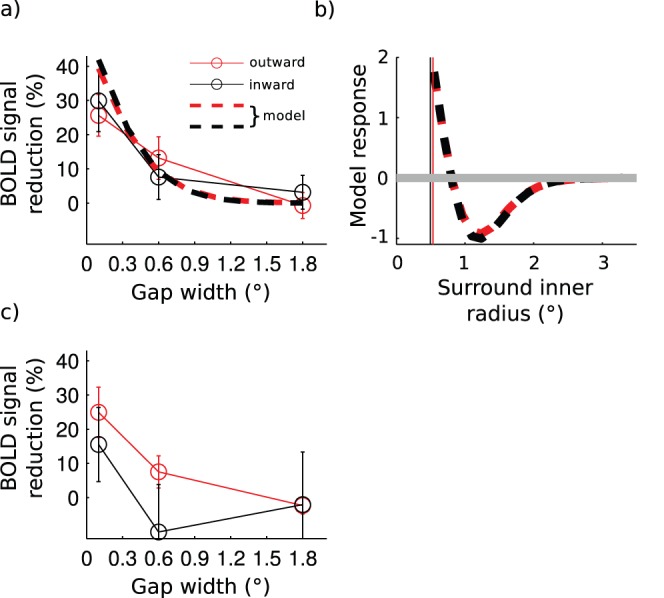
V1 BOLD signal reduction as a function of the gap size. a) Results of the voxel-of-interest analysis. Connected data points mark the mean ± s.e.m. averaged over seven subjects. Dashed lines present suppression strength in the modeled neuronal population in which all parameters were fixed based on the area summation experiment. b) Modeled responses in the voxel-of-interest for surround stimulus displayed without the center. The gray horizontal line marks the baseline response c) Results of the region-of-interest analysis.

The purpose of the first fMRI experiment was to find out whether suppression strength is determined by visual field size rather than cortical size of the surround already at the level of V1. Figure 4a shows the results of the voxel-of-interest analysis. The analysis concerns the signals from a single voxel, situated nearest to the geometric center of the activation produced by the independent functional localizer. When the gap size was small, both the *inward* and the *outward* surrounds strongly reduced the center signal and increasing the gap size decreased BOLD signal reduction. As the gap size of the inward surround was increased from 0.1 to 1.8°, the mean BOLD signal reduction decreased from 29.9±9.0% to 3.1±5.0% (paired two-tailed t-test, t(6) = 3.48, p<0.05). For the *outward* surround condition, the corresponding decrease was from 25.6±9.0% to −0.7±3.8% (t(6) = 3.13, p<0.05). The BOLD signal reduction did not differ statistically significantly between the inward and the outward surrounds at any of the gap sizes (paired two-tailed t-tests, gap 0.1°, t(6) = −0.45, p = 0.67; gap 0.6°, t(6) = 0.86, p = 0.42; gap 1.8°, t(6) = −0.64, p = 0.55).

The model results were highly similar to the fMRI data (dashed lines in Figure 4a), with *all parameters fixed* by the area summation experiment. The model produced strong signal reduction with small gap sizes and the signal reduction decreased as the gap size was increased. The modeled signal reductions were similar for the inward and the outward surrounds.

The match between the model and the fMRI data indicates that spatial aspects of surround modulation are fairly similar in psychophysics and fMRI after the retinotopic coverage of the voxel has been accounted for. However, our previous study [24] showed that suppression is much stronger in fMRI compared to the spiking output of V1 neurons and therefore the modeled BOLD signal reduction was scaled by 2.7 in this study (see METHODS). Thus, surround modulation strength is not directly comparable between psychophysics and fMRI.

Direct comparison of the modulation strength in fMRI and psychophysics is further complicated because the surround necessarily evokes BOLD signals also in the retinotopic representation of the center [29] and some of these signals may not contribute to surround suppression as measured psychophysically. Interestingly, our model produced clear responses in the modeled voxel when the surrounds were presented without the center (Figure 4b). When the inner edge of the surround was abutting the modeled voxel, the surround produced a strong positive signal. In Figure 4b the vertical lines mark the distance of the modeled voxel edges from the center-point of the stimulus, measured along the horizontal meridian. Strength of the positive signal decreased as the inner radius of the surround was increased and negative signals were observed when the inner radius exceeded ∼0.8°. The peak negative signal occurred at ∼1.25° and further increasing the inner radius decreased the amplitude of the negative signal, which returned to baseline at approximately 2.5° inner radius. These modeling results are well in line with the literature concerning negative BOLD responses [49], [50], [51]. To explicitly address the effect of surround in the retinotopic representation of the center, we measured surround alone responses in the fMRI experiment 2.

Figure 4c shows the results of a conventional region-of-interest analysis, in which the signal changes are averaged from multiple voxels (9.5±1.7 voxels (mean ± s.e.m.), n = 7). As in the voxel-of-interest analysis, BOLD signal reduction was strongest with the smallest gap size and decreased with increasing gap size. The inward surround produced approximately 10% BOLD signal increase when the gap size was 0.6°, and with 1.8° gap neither inward nor outward surround produced significant BOLD signal reduction. Moreover, the difference between the surrounds was not significant in any of the gap sizes (paired two-tailed t-test, gap 0.1°, t(6) = 1.42, p = 0.21; gap 0.6°, t(6) = 1.68, p = 0.15; gap 1.8°, t(6) = −0.02, p = 0.99). The model predictions are not shown together with the region-of-interest analysis, because we did not have the visual field locations for each voxel. These locations are necessary for computing the model output.

### fMRI Experiment 2: Cortical Mechanisms of Symmetric Surround Suppression

In standard single unit recordings the modulatory surrounds do not, by definition, produce spikes from the recorded cell [9] and thus this method has not been used for studying the signals that the surrounds produce when presented alone. However, it is expected that the surrounds alone produce sub-threshold neural signals [52] as well as BOLD signals in the center region [53] and studying these signals may help in understanding why surrounds with highly differing cortical representations nevertheless produced similar suppression in the psychophysical experiment and in the first fMRI experiment. Was the suppression similar simply because the surrounds produced similar signals in the cortical representation of the center or were there more complex mechanisms involved? Therefore, to better understand why the two surrounds produced equally strong suppression, we investigated the signal strengths that the surrounds produced in the center region-of-interest when presented alone and when combined with the center. Importantly, the combined signal necessarily contains contributions also from the surround.

**Figure 5:**
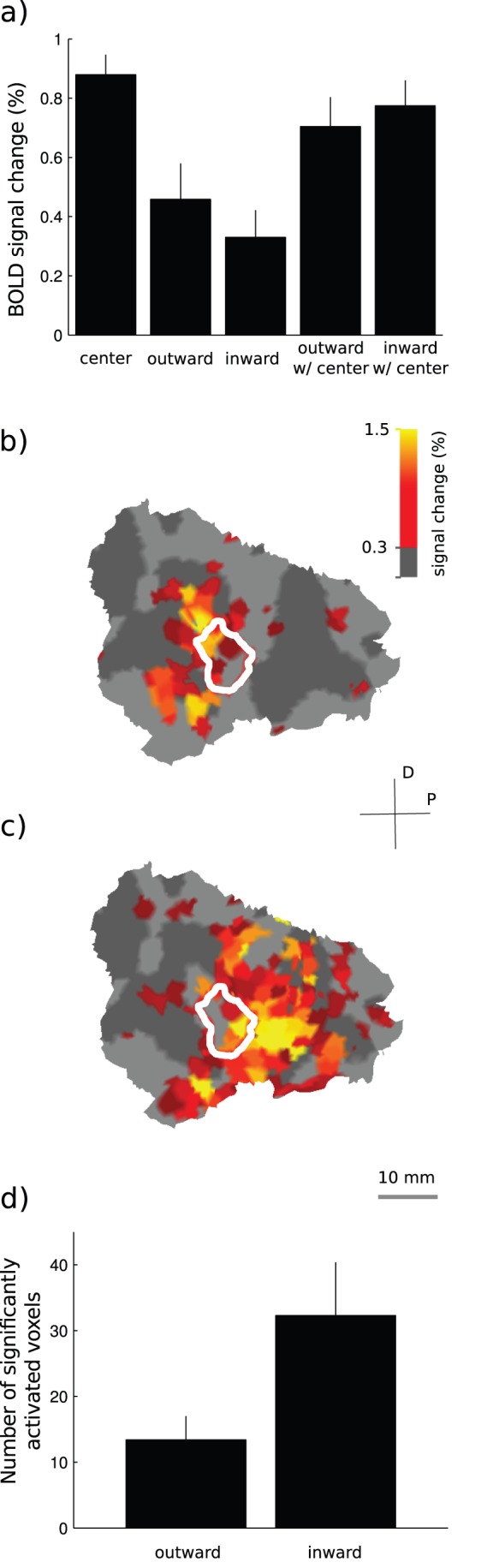
Responses in the center region-of-interest. a) The mean ± s.e.m. of the BOLD signal changes averaged over 10 subjects. b) Outward surround alone signals for one subject projected to the unfolded surface of primary visual cortex. The white continuous curve marks the area activated by the center alone localizer with 100% contrast. c) Same as b but for the inward surround. d) Number of significantly activated voxels in the surround only conditions.

Figure 5a shows the BOLD signals that the center, the *inward* and the *outward* surrounds alone and combined with the center produced in the center region-of-interest. All of these stimuli evoked signals in the center region-of-interest that deviated statistically significantly from zero (one-sample t-test). The BOLD signal changes were 0.88±0.07% (t(9) = 12.9, p<0.001) for the center, 0.33±0.09% (t (9) = 3.58, p<0.01) for the *inward* surround alone and 0.46±0.12% (t(9) = 3.78, p<0.01) for the *outward* surround alone. The *inward* surround combined with the center produced 0.77±0.09% (t(9) = 9.02, p<0.001) BOLD signal change and the *outward* surround combined with the center 0.70±0.10% (t(9) = 7.07, p<0.001). The BOLD signal change was significantly lower when the *outward* surround was combined with the center, compared to the center presented alone (paired one tailed t-test, t(9) = 2.01, p<0.05). The same test for the center alone versus *inward* surround combined with the center was not statistically significant (paired one tailed t-test, t(9) = 1.23, p = 0.13). The BOLD signal change difference between the *inward* and *outward* surrounds was not significant neither when the surrounds were combined with the center (paired two-tailed t-test, t(9) = 0.98, p = 0.35) nor when they were presented alone (paired two-tailed t-test, t(9) = 0.87, p = 0.41).

Figures 5b and c show the surround alone signals from a single subject projected to the unfolded cortical surface. As expected, the number of significantly activated (t-test, family-wise error corrected p<0.05) voxels was larger for the inward than for the outward surround. In the primary visual cortex, the *inward* surround activated on average 32.3±8.10 (mean ± s.e.m) above threshold voxels and the *outward* surround activated on average 13.4±3.61 voxels (Figure 5c). The difference was statistically significant (paired t-test, t(9) = 3.44, p<0.01).

**Figure 6:**
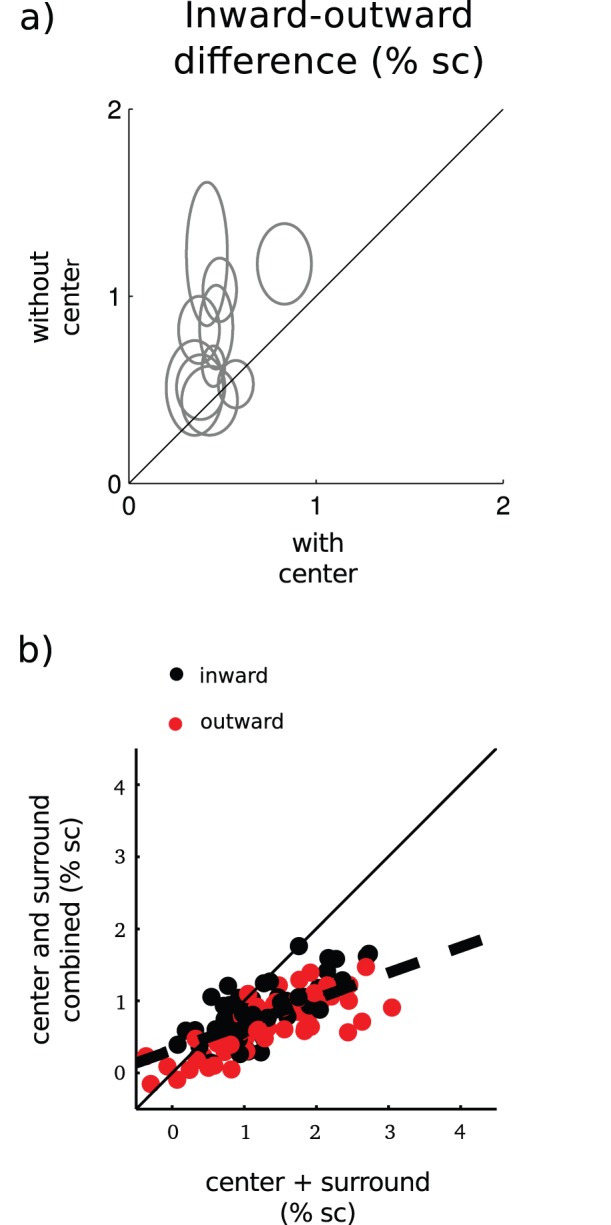
Voxel-wise analyses. a) Center-point of each ellipse marks the mean absolute difference in BOLD signal between the *inward* and the *outward* surrounds averaged over the region-of-interest. Ellipse width marks the corresponding s.e.m. b) BOLD signal change to the simultaneous presentation of the center and the surround as a function of the sum of the center and surround alone signals in the center region-of-interest.

Retinotopic organization of the primary visual cortex suggests that each voxel represents slightly different location of the visual field. Therefore, it is expected that at the level of individual voxels the two surrounds produce slightly different signals. Our data shows that this is indeed the case, but interestingly, the differences were clearly smaller when the surrounds were presented with the center than without the center. Figure 6a shows the absolute difference in BOLD signal strength between the inward and the outward surrounds, without the center (y-axis) and with the center (x-axis). The differences were significantly larger when the surrounds were displayed alone compared to the simultaneous presentation with the center (paired t-test, t(9) = 3.58, p<0.01).Thus, the center-surround interactions attenuated the *inward-outward* differences. Importantly, because the analysis was confined to the voxels in which the response to the simultaneous presentation of the center and the surround was less than to the center alone, saturation of the BOLD response cannot underlie these findings.

Next we will describe how the center-surround interactions attenuate the *inward*-*outward* differences. First, Figure 6b shows that BOLD response to the simultaneous presentation of the center and surround deviated from the un-weighted sum of the center and surround signals. On average, this was well described as a weighted sum of the center and surround responses (dashed line in Figure 6b). This indicates that the attenuation depends linearly on center+surround signal strength. The slope and intercept of the regression line were 0.36 and 0.32, respectively. Second, the attenuation can be defined as 0≤ |*CS_inward_ − CS_outward_*|<|*S_inward_ − S_outward_*|, in which | | denotes absolute value, *S* response to the surround alone and *CS* denotes response to the simultaneous presentation of center and surround. We showed that BOLD response to the combined center-surround stimulus can be described as a weighted sum of the responses to the center and surround alone (Figure 6b). Replacing *CS_inward_* and *CS_outward_* by the corresponding weighted sums *k*(*C+S_inward_*) and *k*(*C+S_outward_*), in which *C* denotes response to the center alone, shows that attenuation follows when *k* is between zero and one.

## Discussion

The purpose of this study was to find out whether strength of surround modulation depends on the visual field size or cortical size of the surround. We found that both in perceived contrast and in V1 BOLD responses the visual field size determined suppression strength. Both of these results were captured by a population of modeled neuronal responses with experimentally fixed parameters. The fMRI results suggested that the surrounds produced equally strong suppression primarily because they evoked signals of equal magnitude in the retinotopic representation of the center. This is surprising because the surrounds activated a significantly different number of voxels in the primary visual cortex. In addition, we found that the signal strength differences between the i*nward* and *outward* surrounds were clearly smaller when the surrounds were combined with the center compared to when they were displayed alone.

### Visual Field Size of the Surround Determines Suppression Strength

The main finding of this study was that two surrounds with markedly different cortical representations, but identical visual field sizes, produced equally strong suppression of both perceived contrast and V1 BOLD responses. Earlier, Petrov et al. [37] reported similar results to the current ones with near threshold targets. However, it was possible that their findings stemmed from ceiling effect because Petrov et al. used six wavelengths surround width and it is known that suppression strength saturates at this width [38]. We controlled for ceiling effects and found that the *inward* and the *outward* surrounds indeed produced suppression of equal strength. The current results clearly show that the visual field size of the surround determines suppression strength, which suggests that the human visual system compensates for the cortical magnification in surround suppression.

### Cortical Mechanisms

According to the human V1 magnification factor [54], the largest gap width in this study (2.1 degrees) translates to approximately 5 mm cortical distance for the *outward* and 12 mm for the *inward* surround. Despite of this 2.4-fold difference in the width of the gap on cortical surface, the *inward* and the *outward* surrounds produced suppression of the same strength. Clearly, a suppressive mechanism which symmetrically extends the same cortical distance towards the fovea and periphery cannot account for this finding. Inactivation experiments [55] and experiments combining anatomical and physiological techniques [7] suggest that both horizontal connections and feedback from extra-striate areas mediate surround modulation in V1. However, the feedback projection cannot fully account for the current findings, because it is asymmetric in the visual field [7]. Quite interestingly, Angelucci et al. [7] reported that the horizontal connections extend over longer cortical distances towards the foveal than the peripheral representation of the primary visual cortex. In fact, if translated to visual field distances, the horizontal connections extend almost exactly the same distance towards the foveal and peripheral visual field [7]. Although it has become increasingly evident that horizontal connections cannot account for the full range [7], [17] and temporal dynamics [56] of surround modulation, recent modeling work suggests that horizontal connections nevertheless contribute to the contextual effects [33]. Our results seem puzzling in light of the known anatomy, because the visual field symmetry of the suppression seems to match with the properties of horizontal connections whereas spatial range of the effects is clearly beyond the mono-synaptic reach of the horizontal connections. An interesting possibility is that the different gap sizes in the *inward* and the *outward* surround conditions would be compensated by their different sized cortical representations thereby leading to visual field symmetry of surround suppression.

We found that despite the markedly different sized cortical representations of the *inward* and the *outward* surround they produced on average equally strong signals at the retinotopic representation of the center when displayed alone. This suggests that the connections underlying surround modulation are organized in such way that surrounds produce equally sized signals when their visual field sizes match. While it is, in principle, possible that the surround alone signals represent purely hemodynamic spreading without any correspondence to neural signals, this is probably not the case. Firstly, it is known that BOLD signals correspond to neural signals even in regions of the primary visual cortex which represent unstimulated parts of the visual field [49]. Secondly, the BOLD signal reflects synaptic inputs more than action potentials [57] and a much larger region of visual field drives synaptic responses than action potentials in V1 neurons [58]. Thirdly, the point-spread of BOLD signals as measured with spin-echo EPI in 3T [41] closely matches the point-spread of sub-threshold electrical neural signals as measured with voltage sensitive dye imaging [59] suggesting that the spread of BOLD signals is not entirely independent of the spread of the electrical neural signals. In line with our findings, Haak, Cornelissen and Morland [53] showed that a V1 voxel with centrally located population receptive field responded to peripheral stimulation when central parts of the stimulus were masked. Their results were neatly captured by a model in which feedback signals from extra-striate cortices underlay BOLD responses in the voxels representing the unstimulated regions of the visual field. It may well be that the surround alone signals that we measured at the retinotopic representation of the center reflect such sub spike-threshold neuronal inputs from the surrounds.

We found that the two surrounds could produce slightly different signal strengths in the individual voxels. This is in harmony with the retinotopic organization of the primary visual cortex. Each voxel represents different visual field locations, which may be closer to either of the surrounds, and therefore in each voxel the surrounds may elicit signals of different strength. What was striking is that these differences were clearly attenuated when the surrounds were combined with the center (Figure 6a). Our analyses showed that the attenuation resulted from the BOLD response to the simultaneous presentation of center and surround being a weighted sum of the component responses with the coefficient between zero and one.

Vanni and Rosenström [60] showed that the BOLD response to a simultaneous presentation of multiple objects is well approximated by a weighted sum of the component responses with the coefficient typically between zero and one. The measured coefficient value in Vanni and Rosenström [60] was in agreement with a prediction which was based on the spatial correlation between the component response patterns. It has been proposed that center-surround interactions remove correlations between component responses in fMRI [60] as well as in single cells [61], [62], [63]. Theoretical considerations suggest that such correlations affect visual coding [64], [65] and thus controlling the correlations is beneficial for information processing in the brain. Because the form of interactions was similar in this study and the study by Vanni & Rosenström [60], it is possible that also the underlying mechanisms are similar.

### Modeling

We used our previously developed model [24] for linking the psychophysical and the fMRI results. Especially considering that all of its parameters were fixed, the model captured both the fMRI and the psychophysical results with a reasonably good accuracy. This result clearly indicates that the spatial characteristics of surround suppression of perceived contrast and V1 responses agree after the retinotopic coverage of a voxel is accounted for. However, the modeled BOLD signal reduction strengths were scaled by 2.7, because strength of surround suppression of V1 BOLD and spike-output differs by this factor [24]. It is not entirely clear to us why the suppression in our previous study was much stronger in fMRI compared to psychophysics, but probably at least some of the discrepancy relates to the BOLD signal reflecting synaptic inputs rather than spikes [57]. The synaptic responses in turn sometimes exhibit stronger suppression than the suppression observed in spike responses [66]. Moreover, the suppression in BOLD responses and in psychophysics is not directly comparable, because the center BOLD response necessarily contains contribution from the surround when the center and the surround are simultaneously displayed (see also discussion above) and it is possible that some of these signals do not contribute to surround suppression as measured psychophysically.

### Surround Modulation Versus Crowding

The current study pertains to the recent discussions concerning similarities and differences between surround modulation and crowding [37], [67]. Crowding is stronger for masks extending towards periphery from the crowded target than for masks extending towards the fovea [37], [68]. The lack of this asymmetry in surround suppression has been one of the key arguments for viewing surround suppression and crowding as distinct phenomena [37], [67]. Our study overcame potential methodological limitations in the Petrov et al. [37] study and confirmed that unlike crowding, surround modulation is symmetric with respect to the fovea-periphery axis.

### Conclusions

Our study demonstrated that although cortical representations of two stimuli would markedly differ, their interactions with other stimuli can nevertheless be highly similar. This suggests that in surround modulation, the human visual system compensates for fovea-periphery anisotropies to better match the statistics of the environment.
